# Commentary: Frontoparietal Structural Connectivity in Childhood Predicts Development of Functional Connectivity and Reasoning Ability: A Large-Scale Longitudinal Investigation

**DOI:** 10.3389/fpsyg.2018.00265

**Published:** 2018-03-01

**Authors:** Wei He, Robert A. Seymour

**Affiliations:** ^1^Department of Cognitive Science, Macquarie University, Sydney, NSW, Australia; ^2^Australian Research Council Centre of Excellence in Cognition and Its Disorders, Macquarie University, Sydney, NSW, Australia; ^3^Aston Brain Centre, School of Life and Health Sciences, Aston University, Birmingham, United Kingdom

**Keywords:** fMRI, MEG, functional connectivity, structural connectivity, development, longitudinal, reasoning

Patterns of functional connectivity (FC) in the human brain are constrained by the structural connections between disparate brain areas (Honey et al., 2009). These structure-function links strengthen with age and have been proposed to underlie the development of diverse cognition and behavior during childhood and into adolescence (van den Heuvel et al., 2015). Yet, little is known about the causal lead-lag relationship between structural connectivity (SC) and FC in supporting the development of high-level cognitive functions.

To address this question in relation to the development of reasoning ability, a recent study by Wendelken et al. (2017) examined the lead-lag relationship between SC and FC using data from 532 individuals aged 6–22 years. Previous work by the authors revealed that two key nodes in the fronto-parietal network, i.e., the rostrolateral prefrontal cortex (RLPFC) and the inferior parietal lobule (IPL), are highly related to reasoning performance in adults (Wendelken et al., 2012). Moreover, FC between RLPFC and IPL has been found to correlate with reasoning development in adolescence (Wendelken et al., 2015).

In their recent study, Wendelken et al. (2017) added DTI/SC measures to the fMRI/FC and matrix reasoning scores. Using cross-sectional data from three large-scale studies, the authors firstly examined the concurrent relationship between the lateral fronto-parietal SC/FC organization and reasoning ability using mixed model regression. They found that the development of reasoning ability reached its peak at 6 years, followed by SC at 7 years, and lastly FC at 13 years. SC between the RLPFC and IPL within fronto-parietal tracts was found to be associated with better reasoning ability in children, and FC between the RLPFC-IPL was related with concurrent increases in reasoning ability only in adolescents and young adults. No significant SC-FC relationship was found at any single time point.

Subsequently, the authors assessed whether SC and/or FC would predict changes in reasoning ability within a smaller longitudinal cohort using a step-wise linear regression. Results suggested that SC predicted FC changes in the fronto-parietal network, but no driving effect of FC was found. Of particular interest is the finding that the SC but not the FC appeared to be a positive predictor for future changes in reasoning ability of children under 12. Together, these results indicate that although both SC and FC between the RLPFC-IPL are significantly related to the development of reasoning ability at different time points, it is the stronger RLPFC-IPL SC during middle childhood that determines the subsequent development of RLPFC-IPL FC and reasoning ability.

Given that FC reflects ongoing neuronal communication underlying cognitive processing, it is surprising that Wendelken and colleagues found that RLPFC-IPL FC could not predict the development of reasoning skills within the longitudinal cohort. We suspect the lack of causal effect of FC might be due to the strong focus on the connectivity between the RLPFC and IPL in the fronto-parietal network. There is growing evidence that other fronto-parietal regions, such as the anterior cingulate cortex, together with the RLPFC and IPL, form a so-called “multiple-demand” system (Duncan, 2010), which gives rise to reasoning ability during complex tasks (e.g., Latin Square Task used in Hearne et al., 2017). Computationally, these regions flexibly interact with each other in a rapid and goal-directed fashion to provide adaptive task control in a wide range of contexts (Cole et al., 2013). On a related note, whilst the authors elegantly described connectivity within the fronto-parietal network, they overlooked other network connections that may as well serve as potential predictors of reasoning development. Reasoning behavior in adults has been found to depend on the efficiency of FC within distributed neural circuits, including the fronto-temporal, cingulo-opercular, and default-mode networks (Finn et al., 2015; Hearne et al., 2017). Therefore, we suggest that future research should assess flexible intra-network connectivity mediated by key areas in the fronto-parietal network, and more importantly should quantify inter-network processes in order to determine the exact neurocognitive architecture underlying the development of reasoning.

Another important consideration, from a more technical point of view, is the implementation of other neuroimaging modalities (e.g., magnetoencephalography, MEG), which can provide dynamic temporal information about the role of fronto-parietal regions in reasoning tasks, into this lead-lag approach. MEG has the ability to track neuronal oscillations in specific frequency bands, which have been linked to high-level cognitive operations (Buzsáki and Draguhn, 2004). For example, increases in the power and coherence of frontal theta-band oscillations (4–7 Hz) are associated with a range of higher-level cognitive control and reasoning tasks (Cavanagh and Frank, 2014). Moreover, this theta activity within the fronto-parietal network has also been shown to predict visual memory performance in children (Astle et al., 2015). By combining high temporal resolution with increasingly sophisticated source estimation techniques, MEG can offer valuable insights into how oscillatory network connectivity for example between fronto-parietal regions could predict reasoning ability in the developing brain (Barnes et al., 2016).

Lastly, we would like to highlight the need to bridge multivariate descriptors of brain development, such as SC-FC coupling, with a richer set of assessments of reasoning behavior. For example, quantitative models combining deductive, inductive, and probabilistic aspects of reasoning (Johnson-Laird and Khemlani, 2013) could be incorporated as multivariate parameters into network neuroimaging data. In this way, future neuroimaging work could go beyond correlations with univariate behavioral indexes (e.g., raw scores of matrix reasoning) and link multifaceted cognitive models of reasoning with SC/FC measures.

In conclusion, Wendelken et al. (2017) demonstrate that the SC between RLPFC and IPL predicts the subsequent development of both RLPFC-IPL FC and reasoning ability. We propose that future neuroimaging work taking a similar developmental perspective could benefit from a brain-wide network based analysis, combined with temporal-scale descriptors of FC measured by MEG and comprehensive, multivariate behavioral models of reasoning.

## Author contributions

WH conceived of the initial idea for this commentary. Both WH and RAS made substantial intellectual contributions to the manuscript and approved the final versions for publication.

### Conflict of interest statement

The authors declare that the research was conducted in the absence of any commercial or financial relationships that could be construed as a potential conflict of interest.
